# Defining Seasonal Windows of Transmission: A High-Resolution Monthly Risk Map for *Leishmania infantum* to Optimize Control Strategies in Southwestern Europe

**DOI:** 10.3390/ani16213463

**Published:** 2026-11-02

**Authors:** Elena Infante González-Mohino, Alfonso Balmori-de la Puente, Manuel Collado-Cuadrado, Alberto Gil-Abad, Elena Carretón, Rodrigo Morchón, Iván Rodríguez-Escolar

**Affiliations:** 1 Zoonotic Diseases and One Health GIR, Biomedical Research Institute of Salamanca (IBSAL), Centre for Environmental Studies and Rural Dynamization (CEADIR), Faculty of Pharmacy, University of Salamanca, 37008 Salamanca, Spain; elena.igm4@usal.es (E.I.G.-M.); ivanrodriguez@usal.es (I.R.-E.); 2 Internal Medicine, Faculty of Veterinary Medicine, Research Institute of Biomedical and Health Sciences, University of Las Palmas de Gran Canaria, 35413 Las Palmas de Gran Canaria, Spainelena.carreton@ulpgc.es (E.C.)

**Keywords:** *Leishmania infantum*, leishmaniasis, *Phlebotomus perniciosus*, ecological niche model, monthly risk maps, Spain, Portugal

## Abstract

Zoonotic leishmaniasis, caused by *Leishmania infantum*, is an emerging public health concern in Southern Europe, where climate change is altering vector phenology. Traditional static risk maps fail to capture critical seasonal transmission dynamics. This study analyzed the monthly risk maps of *L. infantum* infection across Spain and Portugal, including the Balearic, Canary, Madeira, and Azores archipelagos. We developed Ecological Niche Models (MaxEnt) for the primary vector, *Phlebotomus perniciosus*, and integrated them with a temperature-dependent parasite development model to generate high-resolution monthly risk maps. The models exhibited high predictive power. In the Iberian Peninsula, human footprint was the primary driver, revealing a marked seasonal expansion of risk towards northern latitudes and higher altitudes during summer (June–September), with potential transmission windows extending into autumn. Conversely, in Macaronesia, temperature was the dominant factor. The Canary Islands and Madeira showed sustained high risk in coastal areas year-round, while the Azores, historically considered non-endemic, displayed climatic suitability hotspots in central islands. These findings confirm the tropicalization of transmission patterns and identify cryptic risk periods. This dynamic approach offers a robust tool for veterinary and public health authorities to transition from static, calendar-based prophylaxis to adaptive, evidence-based control strategies, optimizing resources in a changing climate.

## Simple Summary

A study based on dynamic models reveals that zoonotic leishmaniasis on the Iberian Peninsula spreads northwards and into higher-altitude areas between summer and autumn. In the Canary Islands and Madeira, the risk along the coast persists throughout the year, whilst outbreaks are emerging in the Azores. The tropicalisation of transmission driven by climate change requires a shift from fixed timetables to adaptive public health control strategies.

## 1. Introduction

Leishmaniasis is one of the vector-borne zoonotic diseases with the highest impact on public and veterinary health worldwide. Caused by protozoa of the genus *Leishmania*, this disease is widely distributed in tropical and subtropical regions, as well as in the Mediterranean basin, where *Leishmania infantum* is the predominant etiological agent [1,2]. As a vector-borne disease, there is a dynamic interaction among its most important hosts, animal reservoirs (mainly the domestic dog), its vectors (phlebotomine sand flies), and humans, representing a significant zoonotic risk [3,4].

In Southern Europe, zoonotic leishmaniasis not only constitutes a veterinary concern due to the severity of the clinical disease in dogs but is also an emerging disease in humans, particularly in immunocompromised individuals, with an increasing number of visceral and cutaneous leishmaniasis cases reported in recent decades [4]. Currently, there is a geographical expansion largely attributed to climate change, which is altering the phenological patterns of the vectors. Rising global temperatures are prolonging vector activity periods, advancing their emergence in spring, delaying their diapause in autumn, or even extending activity throughout the year. This phenomenon increases the risk of infection in both established endemic areas and new foci of emergence [5,6,7].

Spain and Portugal are considered endemic countries for animal leishmaniasis, with significant upward trends in the last decade. Traditionally, the disease was restricted to the Mediterranean basin and the central–southern zone of the Iberian Peninsula; however, it is expanding towards northern latitudes and higher altitudes, areas historically considered risk-free. In Spain, the national mean seroprevalence of *Leishmania infantum* in dogs ranges between 10.12% and 10.36%. The infection is present throughout the country, with peak rates (>12.5%) concentrated in the southern, eastern, and Balearic regions. In northern communities, overall prevalences are more moderate (ranging between 0.8% and 6.6%), although specific hyperendemic foci exist, such as Ourense province [8,9]. Conversely, in Portugal, the mean seroprevalence ranges between 6.3–21.3% [10,11,12], with Lisbon, the capital, at 13.4%, and the highest seroprevalences reported in the south (16.06–60.4%) [13,14,15,16,17]. In cats, the seroprevalence rates observed in Spain and Portugal are consistent with the prevalence rates recorded in dogs [18,19].

To predict and understand the distribution of the disease and anticipate potential changes, Ecological Niche Models (ENMs) have become essential epidemiological tools. By using Maximum Entropy (MaxEnt) algorithms, it is possible to correlate vector presence records with environmental variables to generate habitat suitability maps. Recent studies have successfully applied these techniques to model the distribution of sand fly vector species in various regions, including Iran, Nepal, and Brazil. Furthermore, these models have been used to analyze the risk of *L. infantum* infection in regions such as Thessaly (Greece), as well as the national level in Spain, Portugal, and Greece, often weighting suitable vector habitat with parasite behavior within the vector—such as the optimal infection rate within the 10–30 °C temperature range [20,21,22,23,24,25,26,27,28,29]. However, most of these studies generate static or annual risk maps which, while useful for identifying general endemic areas, lack the temporal resolution necessary to understand seasonal transmission dynamics. Although some recent research has introduced monthly temporal resolution, such as forecasting *Phlebotomus papatasi* activity in Iran [30], these approaches are often limited to generating binary maps based on temperature thresholds for vector survival or activity (e.g., defining activity periods) rather than integrating a continuous assessment of infection risk and parasite–vector interactions throughout the year.

In Spain and Portugal, several vector species of the disease have been identified, with *Ph. perniciosus* being the most widely distributed and, together with *Ph. ariasi*, among the most competent [31]. The biology of these vectors is intrinsically linked to environmental and climatic factors. Previous studies in the Mediterranean basin, such as those conducted in Greece, Spain, Portugal and in Europe in general, have demonstrated that bioclimatic variables, such as mean temperature and human footprint, determine habitat suitability and vector density due to their significant contribution to the studied models [26,27]. Specifically, *Ph. perniciosus* exhibits great ecological plasticity, capable of colonizing areas ranging from semi-arid zones to humid peri-urban environments, whereas *Ph. ariasi* tends to prefer cooler and more humid areas, dominating in regions of higher altitude or northern latitude [31].

Although the distribution of *L. infantum* in the Iberian Peninsula and the mean annual risk of infection have been analyzed, there are no studies determining the temporal risk of infection. Considering that disease transmission is not a constant phenomenon throughout the year and likely fluctuates month by month depending on climatic conditions allowing vector and parasite activity, such an analysis would help design more precise control strategies needed to refine epidemiological surveillance.

In light of this, our objective was to study the monthly risk of *L. infantum* infection in Spain and Portugal, covering both the Iberian Peninsula and their islands and archipelagos. By integrating updated vector distribution data with high-temporal-resolution climatic variables and the behavior of the parasite within the vector, we aim to provide a dynamic predictive tool. This provides an operational framework to support health authorities and veterinarians in optimizing prophylaxis and vector control calendars, adapting them to the changing reality of each month and geographical region.

## 2. Materials and Methods

### 2.1. Study Area

**Figure 1:**
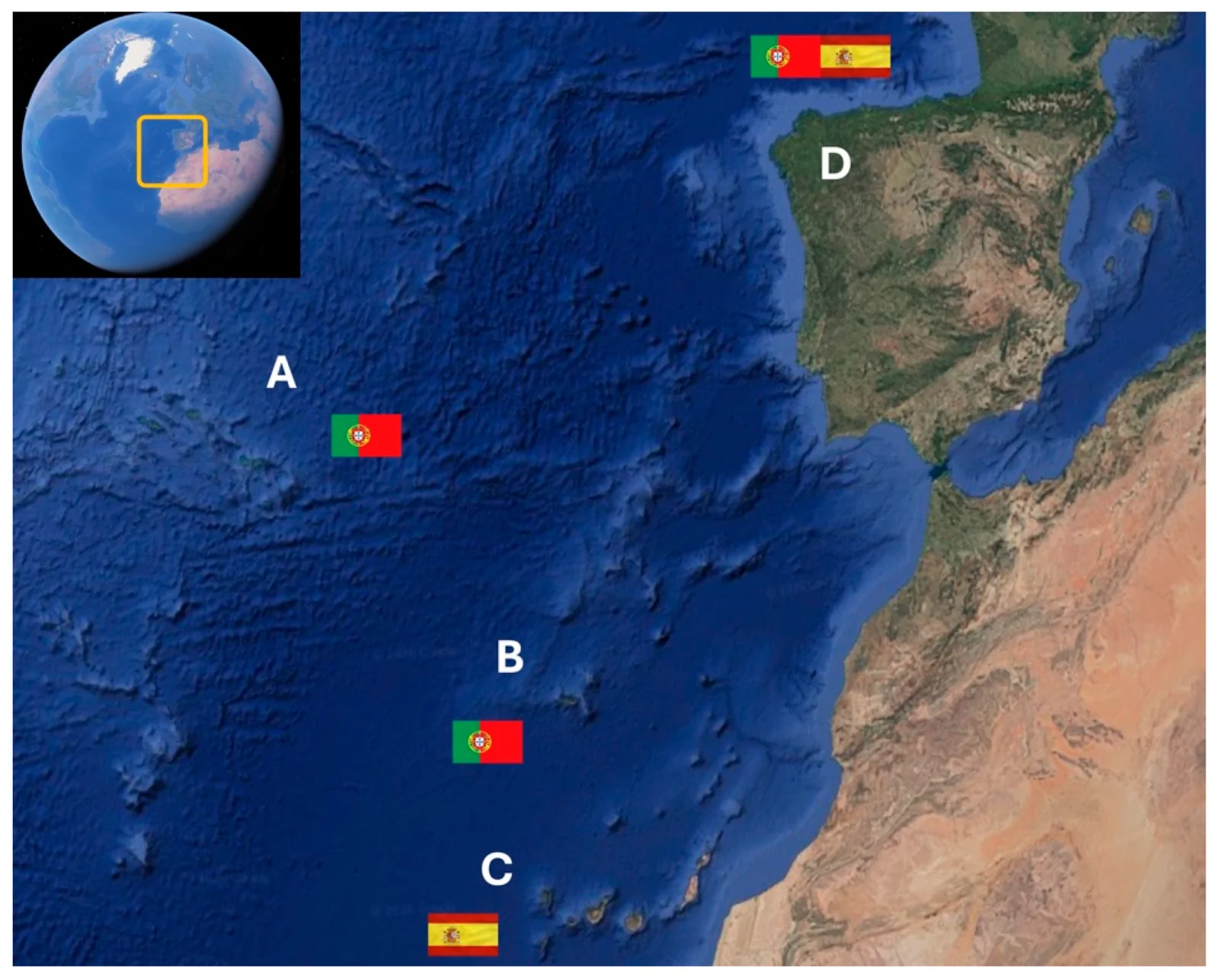
Geographical locations of the Azores (A) and Madeira (B) (Portugal), Canary Islands (C) (Spain), and Iberian Peninsula and Balearic Islands (D) (Spain and Portugal).

The Iberian Peninsula (36°00′–43°47′ N, 9°30′ W–3°19′ E) and the Balearic Islands (Spain) (38°38′–40°06′ N, 1°12′–4°19′ E) have been established as study areas, as well as the Macaronesian archipelagos that form part of Spain and Portugal: the Canary Islands (Spain) (27°37′–29°25′ N, 13°20′–18°10′ W), Madeira (Portugal) (32°38′–33°07′ N, 16°39′–17°16′ W), and the Azores (Portugal) (36°55′–39°43′ N, 24°46′–31°16′ W) (Figure 1). These territories are the southernmost of the European continent, and this geographical location influences the presence of subtropical climates, unlike the rest of Europe. The peninsular area is surrounded by the Mediterranean Sea, the Cantabrian Sea, and the Atlantic Ocean. The Balearic Islands (Spain) are located in the Mediterranean Sea, and the Canary Islands (Spain), Azores, and Madeira (Portugal) are located in the Atlantic Ocean. The Iberian Peninsula covers an area of approximately 590,000 km^2^, while the Balearic Islands, the Canary Islands, Madeira, and the Azores have areas of 4992 km^2^, 7492 km^2^, 825 km^2^, and 6082 km^2^, respectively. The Iberian Peninsula is crossed by several large and small rivers with large river basins and various mountain ranges. Only in Menorca (Balearic Islands) is there a permanent river, the Cala Galdana on the island of Menorca. In the Azores, there are small streams on the larger islands, while in the Canary Islands and Madeira, the hydrographic network consists of irregular watercourses and ravines, with no rivers as such, all of which are of volcanic origin [32,33,34,35].

According to the Köppen–Geiger climate classification, the Iberian Peninsula has a predominantly warm Mediterranean climate (Csa) in coastal and low-lying areas, with hot, dry summers and mild winters. In Madeira and in the high and inland areas of the peninsula, the Mediterranean climate with mild summers (Csb) predominates, characterized by cooler summers and better distributed rainfall. In the Azores and the north and northwest of the peninsula, there is an oceanic climate (Cfb), with frequent rainfall and mild temperatures. The driest areas in the southeast of the peninsula have semi-arid climates, both cold (Bsk) and warm (Bsh), which are characterized by low rainfall and hot summers. In mountainous areas, the climate is continental, with cold winters and short, cool summers. The Canary Island archipelago has subtropical climates that vary from hot desert to semi-arid, depending on orientation and altitude [36].

### 2.2. Vector Occurence Data

*Ph. perniciosus* is the main vector of canine leishmaniasis in Europe [37,38,39,40]. To create the habitat suitability model for this species, georeferenced vector presence points (confirmed entomological occurrence records) were extracted from published studies [15,31,41,42] and the GBIF database [43] in order to obtain as much distribution data on this species as possible. To avoid possible biases associated with the spatial autocorrelation of the presence data, spatial standardization was performed by superimposing them on a grid with 1 km^2^ cells to obtain one point per grid. This processing of the presence points was performed using QGIS software version 3.34.15 [44].

### 2.3. Bioclimatic and Enviromental Variables

The bioclimatic variables used were taken from the World Clim database with a spatial resolution of 1 km^2^. Nineteen variables related to precipitation and temperature between 1970 and 2000 were downloaded [45]. The final selection of variables for the suitability models was made based on a multicollinearity analysis in R software version 4.4.2 [46] using Pearson’s correlation coefficient. When variables were highly correlated, the choice of which variable to retain was guided by its documented relevance as a predictor of vector occurrence and *L. infantum* transmission risk in previous European niche modeling and field distribution studies [27,28,31]. For the model corresponding to the Iberian Peninsula and the Balearic Islands, the selected bioclimatic variables were the mean annual temperature (BIO_1_), the mean diurnal range (BIO_2_), isothermality (BIO_3_), the mean temperature of the wettest quarter (BIO_8_), annual precipitation (BIO_12_), and the seasonality of precipitation (BIO_15_). In the model corresponding to the territory of Macaronesia, the bioclimatic variables selected were BIO_1_, BIO_2_, BIO_3_, BIO_12_, and BIO_15_, in addition to temperature seasonality (BIO_4_) and precipitation in the driest quarter (BIO_17_). On the other hand, for both models, environmental variables such as shrub and herb density [47] and human footprint [48] were also downloaded, which includes variables that reflect human impact on the ecosystem such as population density, cropland and grazing land, and the built environment. All downloaded variables were processed in QGIS to obtain the same extent, resolution (1 km^2^), and coordinate system (GCS_WGS_1984).

### 2.4. Habitat Suitability Model for Phlebotomus perniciosus

To calculate the potential distribution of the vector, two ENMs were developed independently: one for the Iberian Peninsula and the Balearic Islands, and another for Macaronesia (the Azores, Canary Islands, and Madeira), following the methodology used by Rodríguez-Escolar et al. [26]. Both models were generated using the MaxEnt algorithm [49], a predictive modeling tool based on the maximum entropy principle to estimate the potential distribution of the species of interest using presence data [50]. Prior to model calibration, a partitioned dataset scheme was applied to evaluate model performance and ensure dataset independence. From the total set of occurrence records (n = 3032), duplicate spatial coordinates were removed, and spatial filtering was applied to reduce spatial autocorrelation. A random 10% subset (n = 303) was withheld as an independent validation dataset prior to model fitting. The remaining 90% of the dataset (n = 2729) was used for model calibration and evaluation, partitioned into an 80% training set (n = 2183) and a 20% testing set (n = 546).

The Kuenm package of the R software version 4.4.2 [51] was used to select the best-fitting model. This package generated and identified the best MaxEnt models from a set of candidate models created using different combinations of parameters. For modeling *Ph. perniciosus* in the Iberian Peninsula, 119 candidate models were generated for a set of variables, seven feature class combinations (l, q, p, lq, lp, qp, and lqp) representing the relationship between the species’ presence and the environment (linear, quadratic, and product), and 17 regularization multipliers (from 0.1 to 0.9 and from 1 to 10). For the Macaronesia model, 102 candidate models were generated for a single set of variables, six class combinations (l, q, lq, lp, qp, and lqp), and 17 regularization multipliers. From these candidate models, Kuenm selects the best fit based on three selection criteria: statistical significance (Partial_ROC < 0.05), omission rate (OR = 5%), and model complexity using the corrected Akaike Information Criterion for small sample sizes (AICc ≤ 2). Additionally, the area under the curve (AUC) was evaluated, which allows for estimating the overall predictive capacity of the models, with values close to 1 indicating high performance. From the models that met the three evaluation criteria, the final model was selected based on the average AUC obtained using presence points independent of those used in the calibration. The AUC values were calculated internally by Kuenm using the standard MaxEnt procedure, which is based exclusively on presence records and 10,000 random background (pseudo-absence) points within the study area (default setting). The best-performing models selected met all these criteria simultaneously, including low omission rate, statistical significance, reduced complexity, and high AUC values.

### 2.5. Leishmania Infantum Infection Rate in Phlebotomus perniciosus

The infection rate, understood as the probability of the presence of sand flies infected with *L. infantum*, was estimated by adapting the biological model of Rioux et al. [52] for the vector *Ph. perniciosus*. This calculation was articulated through the following increasing logarithmic function:

y = 0.718 [1 − e (−0.237 (x − 8))]

where y represents the rate of infected vectors and x represents the average temperature. This equation allows us to model infectivity within the critical temperature range of 10 to 30 °C, which delimits the biological viability for the survival and replication of the parasite in the digestive tract of the vector [53]. At the computational level, an R language script was optimized based on the methodology of Rodríguez-Escolar et al. [26,27], into which an iterative loop was integrated for thermal data processing. Unlike previous studies, this script processed daily average temperature records from the CHELSA database [53], which allowed us to derive not only annual averages, but also monthly infectivity rates.

### 2.6. Risk Maps of Leishmania infantum Month by Month

To obtain the risk maps, both for the Iberian Peninsula and Balearic Islands model and for the Macaronesia model, the Raster Calculator tool in QGIS 3.34.15 was used. An equal-weighted arithmetic mean was calculated at the pixel level between the final ecological niche model of *Ph. perniciosus* and the previously calculated monthly estimates of the *L. infantum* infection rate. This methodological approach assigned an equivalent weight (50%) to both variables in both regional models, generating a series of maps representing the spatial distribution of the risk of infection throughout the twelve months of the year.

In order to allow for a consistent comparison between regions and periods, the legend values were standardized for all monthly maps. The absolute maximum value observed during the month of highest risk throughout the study area was designated as “high,” while the minimum value for the month with the lowest risk was labeled as “low.” The midpoint between these extremes was defined as “medium.” These standardized categories were applied in QGIS to facilitate visual interpretation of seasonal dynamics and identification of critical transmission periods in each territory.

## 3. Results

### 3.1. Ecological Niche Models of Phlebotomus perniciosus

Of the 119 candidate models evaluated, the model selected for *Ph. perniciosus* in the Iberian Peninsula and Balearic Islands obtained an AUC of 0.752, with habitat suitability values ranging from 0.0026 (minimum) to 0.8246 (maximum) (Figure S1). The areas with the highest suitability were predominantly located on the south coast and the Mediterranean coastline, as well as in the main urban centers. In terms of variables, the one with the highest percentage contribution was human footprint (38.91%), followed by average annual temperature (19.88%) and average daily range (10.29%) (Table 1). On the other hand, the model developed for the Macaronesia region showed a better fit, with an AUC of 0.899 and suitability indices ranging from a minimum of 0.016 to a maximum of 0.522 (Figure S1). In this regional model, the variable that best explained the distribution of the species was the average annual temperature (43%), followed by the average daily range (13.76%) and the human footprint (11.78%) (Table 2). The Canary Islands archipelago showed the highest suitability levels in the region, with Lanzarote standing out as the island with the lowest values within the group. In Madeira, suitability was highest on the southeast coast, while in the Azores, suitability was generally low, except for specific hotspots in the central group of islands, specifically Pico, Faial, Graciosa, and São Jorge.

### 3.2. Seasonal Dynamics of the Leishmania infantum Infection Rate

The average annual infection rate in mainland Spain and the Balearic Islands shows a heterogeneous spatial distribution, with maximum values on the Mediterranean coast and in the southern region, while higher altitude areas and the northern strip show the lowest levels. In the Macaronesia region, the infection rate shows different patterns between archipelagos. The maximum average values are significantly higher in the Canary Islands and Madeira, while the Azores have the lowest infectivity burden. In the Canary Islands, high-risk areas are more extensive and continuous, in contrast to the fragmentation observed in the other islands, where minimum values are restricted to high-altitude areas (Figure S2).

Analysis of monthly dynamics revealed marked seasonality in the Iberian Peninsula and the Balearic Islands, while a constant thermal progression was observed in the archipelagos (Figures S3–S6). In the Iberian Peninsula and the Balearic Islands, the infection rate begins to be noticeable in March after winter, reaching values of up to 0.58 (58%) in areas bordering the Mediterranean Sea and the southwestern peninsula (Figure S3). With the onset of spring, a progressive and widespread increase is observed throughout the territory, culminating in a summer maximum of 0.7134 (71.34%) during the month of July. It is noteworthy that, in this month, even the minimum values in the least favorable regions rise to 0.1771 (17.71%), indicating a window of global risk. During the fall, the infection rate experiences a gradual decline that progresses from north to south, with the highest values persisting exclusively in the thermal refuges of the coast and the south of the peninsula.

The monthly dynamics in the archipelagos (Figures S4–S6) show a constant thermal progression: the minimum values (0.0582 or 5.82%) are recorded in winter and at high altitudes such as Teide (Tenerife, Canary Islands) and Pico Mountain (Azores). The infection rate rises steadily during the spring until it reaches its peak in August (0.6551). In all the periods analyzed, the highest percentage of infected vectors is located in coastal areas and in areas with higher population density, where environmental conditions favor the stability of the biological cycle.

### 3.3. Maps of Risk of Infection by Leishmania infantum

The average potential risk of infection by *L. infantum* is shown in Figure S7. There is a high risk of infection in the southern part of the peninsula and on the Mediterranean coast, as well as in the Balearic Islands, where the highest values are reached (0.62287). This risk decreases to average values in the central area—where conditions are favorable for the vector—and reaches low and very low values as altitude and latitude increase towards the north (minimum of 0.15995). On the other hand, in the Canary Islands, a high risk is observed in coastal areas and urban centers (maximum of 0.51904), while Madeira has constant medium-high values (0.39744). As on the peninsula, the risk drops dramatically in high mountain areas, as observed at the summit of Teide (Tenerife, Canary Islands) and Pico Mountain (Azores) (minimum of 0.2759).

**Figure 2:**
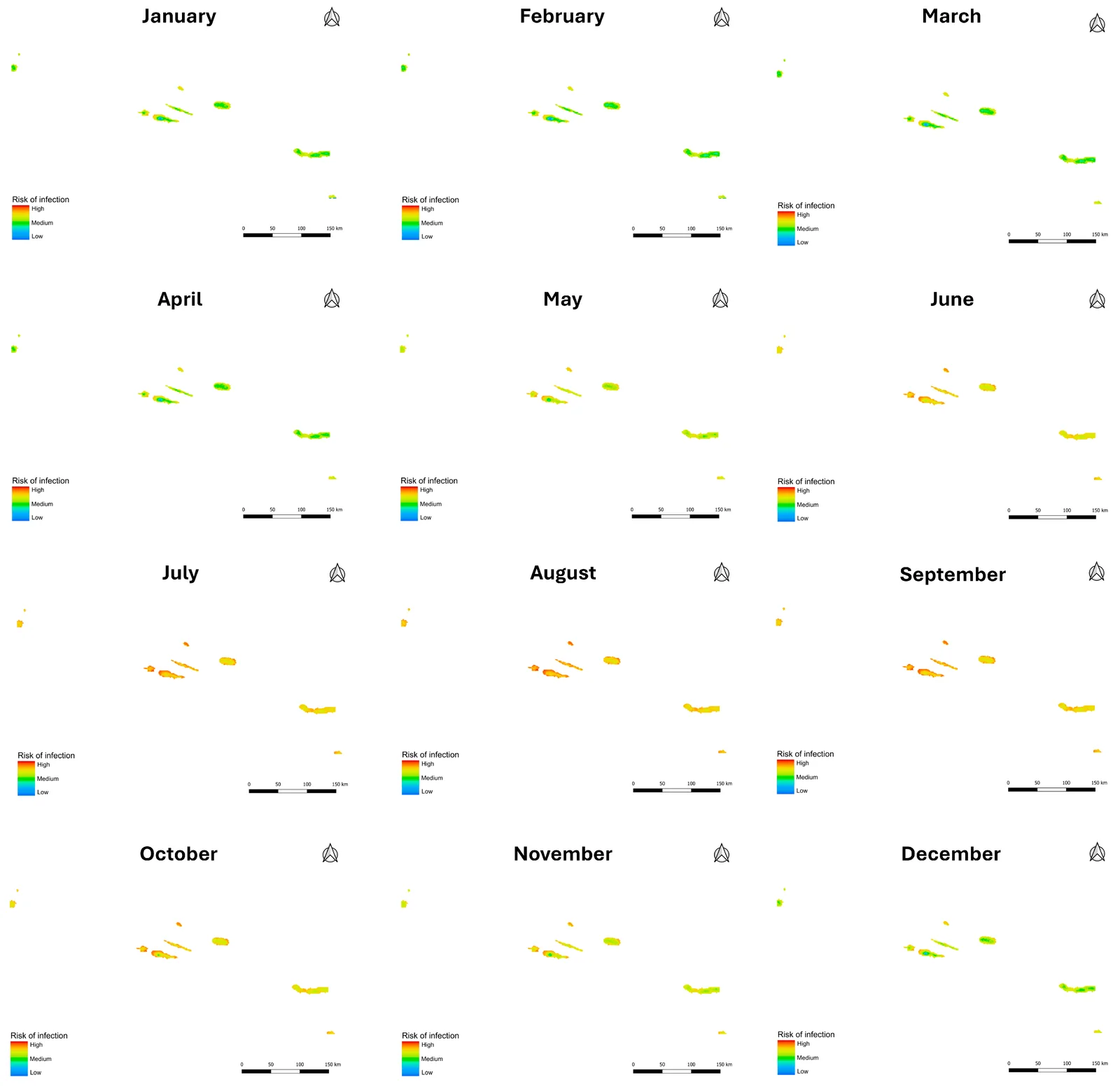
Monthly infection risk maps for *Leishmania infantum* in Azores (Portugal) generated by combining the ecological niche model of 100% *Phlebotomus perniciosus* suitability with the estimated monthly infection rate of parasite.

The monthly spatiotemporal analysis of the risk of infection by *L. infantum* in mainland Spain and Portugal (Figure 2) showed marked seasonality, with the risk remaining at minimal levels (0.001256), where the winter months show a predominance of low overall risk, restricting potential transmission almost exclusively to southern coastal areas. However, coastal and southern areas maintain moderate values. As the year progresses, the risk expands latitudinally, reaching its maximum intensity and geographical distribution between June and September, with high-risk areas extending over much of the territory, with August being the month with the highest risk of infection (0.76748). Finally, the annual cycle concludes with a progressive retraction of risk areas during the fall, returning to minimum levels around December, although certain high-altitude areas remain unsuitable throughout the period.

**Figure 3:**
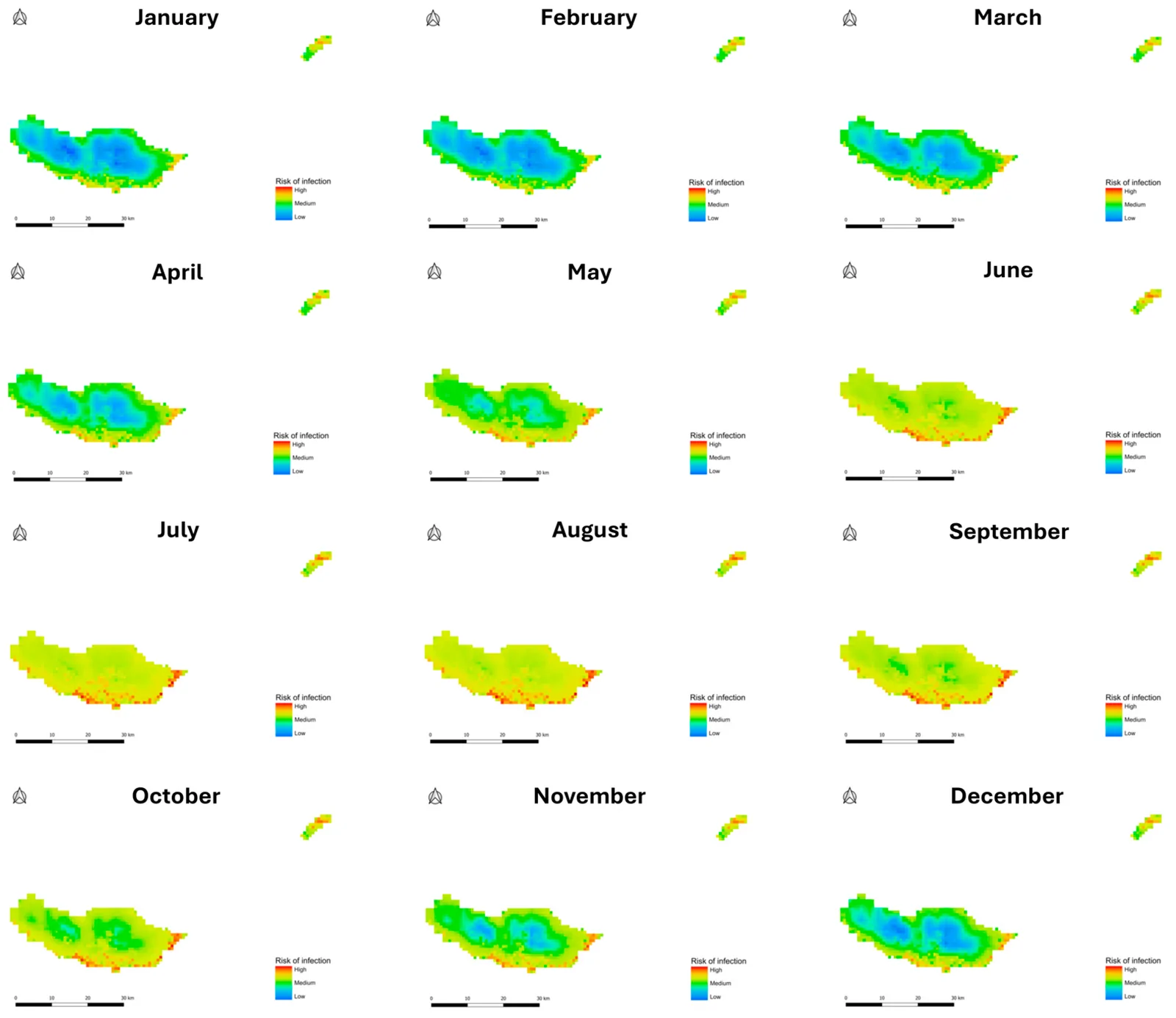
Monthly infection risk maps for *Leishmania infantum* in Madeira (Portugal) generated by combining the ecological niche model of 100% *Phlebotomus perniciosus* suitability with the estimated monthly infection rate of parasite.

**Figure 4:**
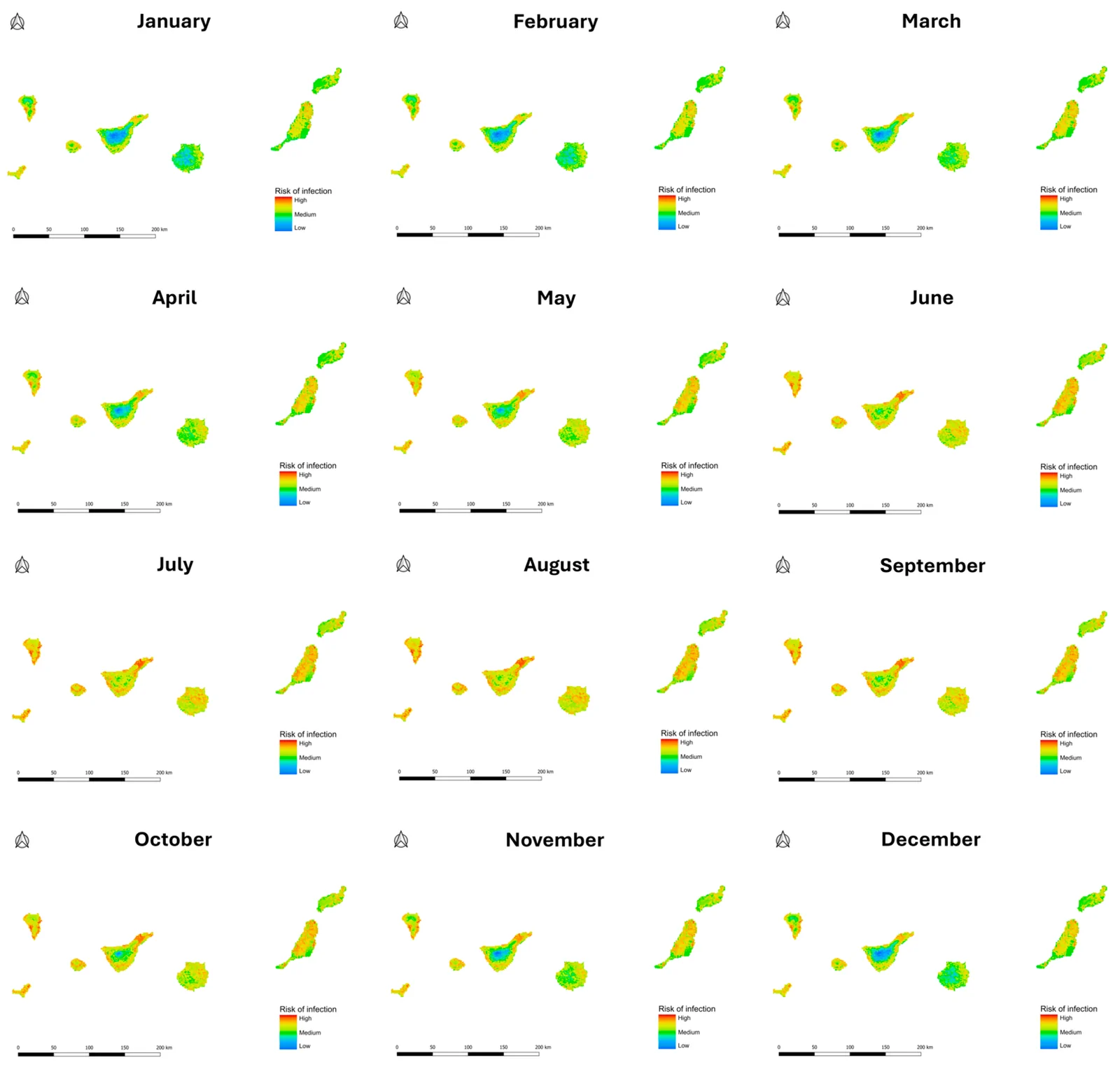
Monthly infection risk maps for *Leishmania infantum* in Canary Islands (Spain) generated by combining the ecological niche model of 100% *Phlebotomus perniciosus* suitability with the estimated monthly infection rate of parasite.

**Figure 5:**
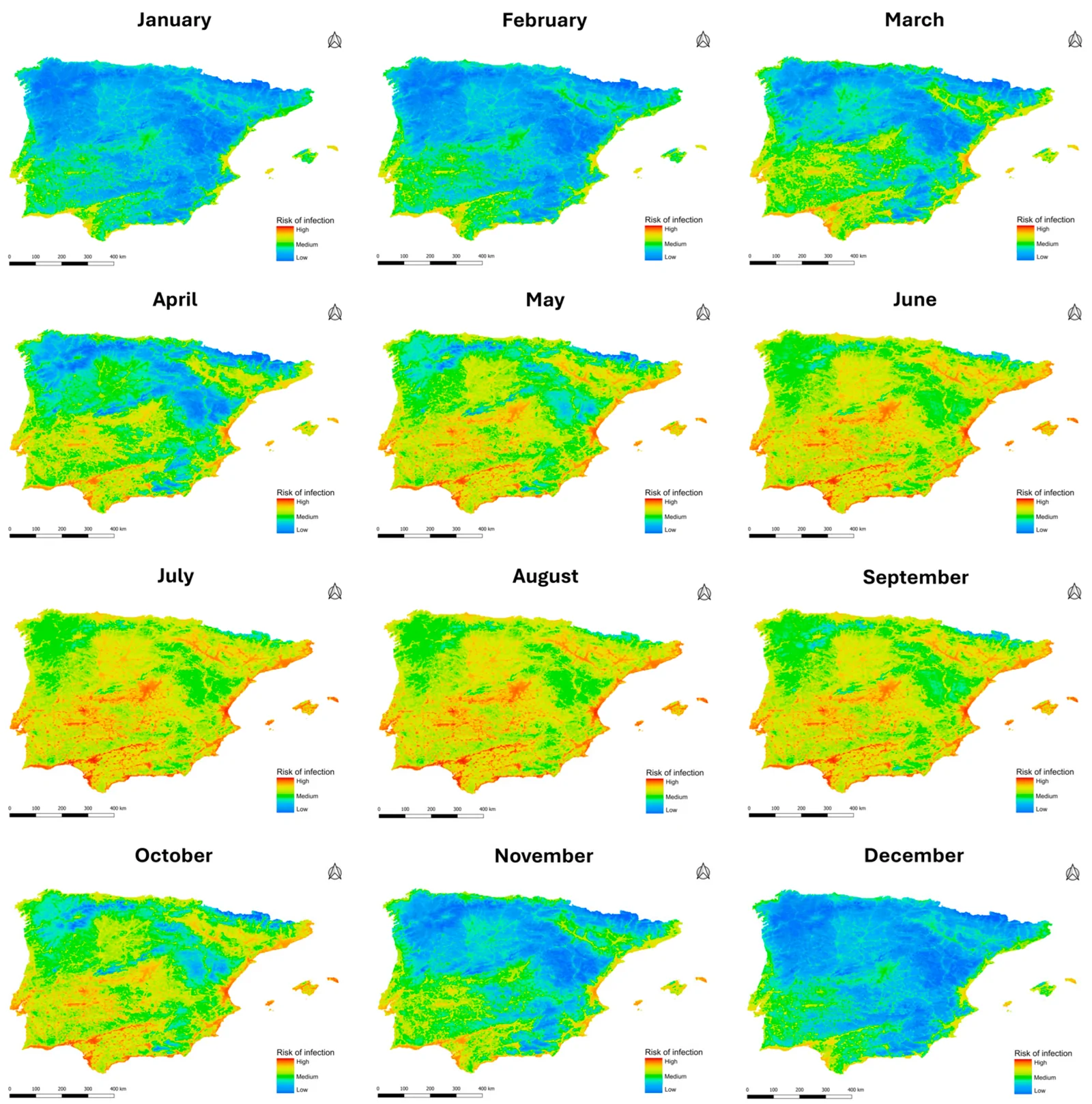
Monthly infection risk maps for *Leishmania infantum* in Iberian Peninsula and Balearic Islands (Spain and Portugal) generated by combining the ecological niche model of 100% *Phlebotomus perniciosus* suitability with the estimated monthly infection rate of parasite.

Meanwhile, in the Macaronesia archipelagos (Figure 3, Figure 4 and Figure 5), the dynamics behave differently due to the Atlantic influence and the island topography. During the winter months and early spring, climatic suitability remains at low or moderate levels, with risk restricted mainly to coastal areas and higher altitude areas due to thermal barriers in the interior with minimum values (0.0582). As the warm months progress, there is a rapid expansion and intensification of risk, reaching its peak between July and September, a period in which high values predominate in most of the island territory, with the maximum occurring in August (0.6029). Finally, the annual cycle concludes with a gradual and sustained decrease in favorability during the fall, returning in December to a baseline risk scenario confined once again to the more temperate coastal areas.

## 4. Discussion

This study provides the first high-resolution spatiotemporal analysis of *Leishmania infantum* infection risk in southwestern Europe (Spain and Portugal) on a monthly basis in the Iberian Peninsula and Macaronesia (Canary Islands, Madeira, and Azores). While previous studies have successfully employed Ecological Niche Models (ENMs) to predict the static distribution of vectors such as *Ph. perniciosus* [7,26,29], our results demonstrate that static maps, although useful for identifying general endemicity, fail to capture critical temporal transmission windows. The infection risk is not constant in most of the studied territories but fluctuates significantly, revealing risk periods that may go unnoticed. This dynamic methodological approach allows for a precise distinction between vector presence and transmission capability month by month, a critical nuance often overlooked in annual prevalence maps [5].

The ENMs developed in this study exhibited high predictive performance for both the Iberian Peninsula and Macaronesia (Canary Islands, Madeira, and Azores), with AUC values of 0.752 and 0.899, respectively, validating the selection of bioclimatic and environmental variables. A crucial finding is the dominant role of the “human footprint” variable in the peninsular model. This corroborates the characterization of *Ph. perniciosus* as a highly plastic and anthropophilic species, capable of adapting to peri-urban and agricultural environments by exploiting microclimates in human settlements that act as thermal buffers [54,55]. This anthropophilic behavior explains the persistence of high-risk areas in densely populated coastal regions and large urban centers, and consequently with a high number of domestic reservoirs such as dogs, a trend also observed in recent seroprevalence studies [10,12,56]. Conversely, in the Macaronesia region, the hierarchy of ecological drivers reveals distinct dynamics. In these archipelagos, the annual mean temperature (BIO_1_) emerges as the dominant explanatory variable. This suggests that, in the insular environment, the thermal stability provided by the buffering effect of the Atlantic Ocean is the main determinant for vector survival, outweighing landscape modification.

However, the anthropogenic factor is not negligible. Although the climate allows for widespread survival at low altitudes, effective transmission is concentrated in urbanized coastal zones, where host density (dogs and humans) maximizes vector–host contact in a climatically optimal environment throughout the year [28,57,58,59]. This highlights a clear demographic contrast with the mainland, where human population density and land modification are widely distributed, whereas in insular environments human density is strictly localized along narrow coastal fringes. Furthermore, our models confirm globally that temperature acts as the main limiting factor for parasite development within the vector. The 10–30 °C biological window used in our study is supported by experimental evidence demonstrating that *L. infantum* can develop in sand flies even at lower temperatures (20 °C), suggesting a high potential for adaptation to cooler climates [59,60].

Our monthly maps reveal marked seasonal spatial variation in potential transmission risk, with high-risk conditions reaching northern latitudes and higher altitudes during peak summer months, which is consistent with broader observations on environmental suitability for vector-borne diseases in southern Europe [28,61]. Areas in the northern Iberian Peninsula (Spain and Portugal) show favorable transmission conditions during the summer months, aligning with recent entomological surveys that report the presence of Phlebotomus species in areas previously considered vector-free [62,63]. Furthermore, the extension of the risk period into October and November in southern regions aligns with phenological shifts observed in sand fly populations, where shorter winters delay diapause and extend the active season [58,60]. Therefore, disease surveillance should be reconsidered and extended to the northern peninsula.

Regarding the monthly infection risk in the Macaronesian archipelagos, the influence of oceanic buffering and topography on transmission dynamics is noteworthy. In the Canary Islands, Azores and Madeira, the risk is constant and high throughout the year in coastal areas, likely due to thermal stability facilitating continuous vector cycles, a pattern consistent with the hyperendemic status of these archipelagos [11,12,13,14,64]. While these model outputs indicate suitable environmental conditions during certain months, the actual absence of active transmission may depend on a combination of factors, such as the lack of established vector populations, host movement constraints, or limited historical introduction of the pathogen, rather than climatic limitations alone. These findings highlight the importance of targeted entomological and epidemiological field surveys to confirm vector presence and assess potential local vulnerability [61].

However, translating predictive models into public health action in southwestern Europe faces administrative decentralization and political turnover across Spain, Portugal, and island territories. To overcome these hurdles, our findings can be feasibly integrated into existing One Health surveillance networks without requiring new infrastructure. From a regional perspective, northern peninsular authorities can utilize these dynamic maps for targeted summer surveillance, whereas southern and island agencies can optimize extended or year-round vector control and canine reservoir interventions. Standardizing these indicators within routine workflows will help buffer preventive programs against administrative shifts.

This study has limitations inherent to correlative modeling. The use of vector presence data from GBIF may introduce sampling bias, although we mitigated this by applying spatial filtering. Additionally, the model assumes that the presence of *Ph. perniciosus* is the main driver of transmission, potentially underestimating risk in areas where other competent vectors like *Ph. ariasi* are dominant, particularly in cooler and wetter peninsular regions; however, the model is strengthened by using the most abundant and widely distributed species in the analyzed areas. Finally, although the human footprint variable serves as a proxy for reservoir presence, the actual distribution of seropositive dogs was not studied, implying that the realized risk could differ from the environmental risk presented. Furthermore, our seasonal models depict potential environmental transmission risk rather than monthly clinical incidence, as the long and variable incubation period of *L. infantum*, combined with diagnostic delays, variable clinical presentation, and underdiagnosis, creates a temporal disconnect between parasite transmission and clinical case reporting.

## 5. Conclusions

This study demonstrates that the risk of *L. infantum* infection in southwestern Europe is a highly dynamic phenomenon driven by the synergy between climate warming and anthropogenic landscape modification. We identified a clear expansion of risk areas towards the north and higher altitudes, as well as an elongation of the transmission season in endemic areas. The identification of suitable habitats in the Canary Islands, Madeira, and Azores challenges the notion that these archipelagos are permanently risk-free. These monthly risk maps provide a scientifically robust tool for health authorities and veterinarians to transition from static, calendar-based prophylaxis to dynamic, evidence-based interventions, optimizing resources and improving the protection of public and animal health in a changing climate. Although it is difficult to precisely predict future impacts due to the interaction of multiple factors, meteorological surveillance can serve as an early warning system to reduce the risk of epidemic outbreaks.

## Figures and Tables

**Table 1:** Analysis of the contribution of the 9 bioclimatic and environmental variables to the ecological niche models for *Phlebotomus perniciosus* in Iberian Peninsula and Balearic Islands (Spain and Portugal).

Variable	% Contribution
Human footprint	38.91
Annual mean temperature (BIO_1_)	19.88
Mean diurnal range (BIO_12_)	10.29
Annual precipitation (BIO_12_)	8.97
Shrub density	7.07
Isothermality (BIO_3_)	4.31
Herbaceous density	3.87
Mean temperature of wettest quarter (BIO_8_)	2.10
Precipitation seasonality (BIO_15_)	2

**Table 2:** Analysis of the contribution of the 10 bioclimatic and environmental variables to the ecological niche models for *Phlebotomus perniciosus* in Macaronesian model: Azores and Madeira (Portugal) and Canary Islands (Spain).

Variable	% Contribution
Annual mean temperature (BIO_1_)	43.00
Mean diurnal range (BIO_2_)	13.76
Human footprint	11.78
Precipitation of Driest Quarter (BIO_17_)	7.74
Temperature seasonality (BIO_4_)	7.36
Precipitation seasonality (BIO_15_)	6.20
Isothermality (BIO_3_)	5.40
Annual precipitation (BIO_12_)	1.48
Shrub density	1.18
Herbaceous density	0.70

## Data Availability

The raw data supporting the conclusions of this article will be provided by the authors upon request.

## Supplementary Materials

The following supporting information can be downloaded at: https://www.mdpi.com/article/10.3390/ani16213463/s1, Figure S1: Ecological niche models showing habitat suitability for *Phlebotomus perniciosus* in Azores (A) and Madeira (B) (Portugal); Canary Islands (C) (Spain) and Iberian Peninsula; Balearic Islands, Ceuta, and Melilla (D) (Spain and Portugal). Figure S2: Prediction of the infection rate of *Leishmania infantum* in Azores (A) and Madeira (B) (Portugal); Canary Islands (C) (Spain); and Iberian Peninsula and Balearic Islands (D) (Spain and Portugal). Figure S3: Prediction of the monthly infection rate of *Leishmania infantum* in Azores (Portugal). Figure S4: Prediction of the monthly infection rate of *Leishmania infantum* in Madeira (Portugal). Figure S5: Prediction of the monthly infection rate of *Leishmania infantum* in Canary Islands (Spain). Figure S6: Prediction of the monthly infection rate of *Leishmania infantum* in Iberian Peninsula and Balearic Islands (Spain and Portugal). Figure S7: Average infection risk map for *Leishmania infantum* in Azores (A) and Madeira (B) (Portugal); Canary Islands (C) (Spain) and Iberian Peninsula; and Balearic Islands (D) (Spain and Portugal), constructed from the ecological niche model (ENM) of 100% *Phlebotomus perniciosus* suitability and the average infection rate of the parasite within the vector.
